# Verifying the relationships of defect site and enhanced photocatalytic properties of modified ZrO_2_ nanoparticles evaluated by in-situ spectroscopy and STEM-EELS

**DOI:** 10.1038/s41598-022-15557-0

**Published:** 2022-07-04

**Authors:** Hyun Sung Kim, Ye-Jin Kim, Ye Rim Son, Vy Ngoc Pham, Ki-jeong Kim, Chang Woo Kim, Young-Sang Youn, Oh-Hoon Kwon, Hangil Lee

**Affiliations:** 1 Department of Chemistry, Pukyong National University, Busan, 48513 Republic of Korea; 2 Department of Chemistry, Ulsan National Institute of Science and Technology (UNIST), Ulsan, 44919 Republic of Korea; 3 Department of Chemistry, Sookmyung Women’s University, Seoul, 04310 Republic of Korea; 4 Beamline Research Division, Pohang Accelerator Laboratory (PAL), Pohang, 37673 Republic of Korea; 5 Department of Smart and Green Technology Engineering, Pukyung National University, Busan, 48513 Republic of Korea; 6 Department of Chemistry, Yeungnam University, Daehak-ro 280, Gyeongsan, Gyeongbuk 38541 Republic of Korea

**Keywords:** Chemistry, Materials science

## Abstract

Base treatment and metal doping were evaluated as means of enhancing the photocatalytic activity of ZrO_2_ nanoparticles (NPs) via the generation of oxygen vacancies (O_vS_), and the sites responsible for this enhancement were identified and characterized by spectroscopic and microscopic techniques. We confirmed that O_vS_ produced by base treatment engaged in photocatalytic activity for organic pollutant degradation, whereas surface defects introduced by Cr-ion doping engaged in oxidative catalysis of molecules. Moreover, we verified that base-treated ZrO_2_ NPs outperformed their Cr-ion doped counterparts as photocatalysts using in situ X-ray photoelectron spectroscopy and scanning transmission electron microscopy coupled with electron energy loss spectroscopy (STEM-EELS). Thus, our study provides valuable information on the origin of the enhanced photocatalytic activity of modified ZrO_2_ NPs and demonstrates the practicality of in situ spectroscopy and STEM-EELS for the evaluation of highly efficient metal oxide photocatalysts.

## Introduction

In view of their photocatalytic activity, metal oxide nanoparticles (MO NPs; e.g., TiO_2_, CeO_2_, and ZnO) find numerous applications^1–5^. This activity comes from the occurrence of oxygen vacancies (O_vS_) on the NP surface and can therefore be enhanced by increasing the amount of O_vS_, ideally without inducing structural changes. Although the involvement of versatile defect sites in photocatalysis is widely accepted, the distinguishable mechanisms of their actions are not fully understood, largely because direct monitoring of these sites is hindered by their inherent instability^6–11^. The precise control of O_vS_ characteristics through defect structure engineering is widely used to tailor the intrinsic properties of photocatalysts and thus rationally control their photocatalytic degradation (PCD) activity and selectivity^12–14^.

A common method of generating defect sites in MO NPs relies on surface modification via metal doping or base treatment^15–18^. During metal doping, surface defects are formed through charge transfer due to the combination of oxygen in MO NPs and doped transition metal ions. Meanwhile, many OH- groups formed on the surface during base treatment of NP affect the formation of hydroxyl radicals (^**·**^OH) as a formation of O_vS_ which is directly related to the photocatalytic activity^2^. As it is currently unclear which of the two methods is more efficient for photocatalytic activity enhancement, there is a need to identify and characterize photocatalytic-related defect structures directly affecting photocatalytic properties. The generated positive holes in the valence band (VB) react with water and produce ^**·**^OH. As soon as ^**·**^OH is produced, they react with organic compounds and oxidize them, and eventually, CO_2_ and H_2_O as end products are produced^19^.

Contrary to other MOs, ZrO_2_ NPs cannot efficiently utilize visible light because of their wide bandgap (~ 5.0 eV) but show promising physicochemical properties, namely high thermal and chemical stability, low thermal conductivity, high corrosion resistance, and high strength. Accordingly, ZrO_2_ NPs have attracted much attention as multifunctional materials for catalysis, dye-sensitized solar cells, fuel cells, and gas sensors^20–22^. Therefore, this research is also meaningful and can be worth maximizing the photocatalytic activity of ZrO_2_ NPs by generating defect sites on their surface via metal doping or base treatment.

Herein, we verified base treatment and Cr-ion doping in terms of their ability to enhance the photocatalytic activity of ZrO_2_ NPs and determined which method affords defect structures directly responsible for this activity. The defect and electronic structures of three types (pristine, base-treated, and Cr-doped) of ZrO_2_ NPs were probed using high-resolution X-ray photoelectron spectroscopy (HRXPS), X-ray absorption spectroscopy (XAS), and scanning transmission electron microscopy coupled with electron energy loss spectroscopy (STEM-EELS), while photocatalytic activity was assessed by monitoring the degradation of 4-chlorophenol (4-CP) and phenol in aqueous solutions. In addition, the generation of ^**·**^OH over the above photocatalysts was assessed by following the formation of *p*-hydroxybenzoic acid (*p*-HBA) from benzoic acid (BA). For abundant O_vS_, charge carrier activity increases in the external supply of additional energy (i.e., photons with energies exceeding that of the bandgap) during spectroscopic measurements^23–25^. Therefore, in situ XPS measurements were herein performed under the conditions of the PCD reaction to characterize the change of electronic structures produced upon the irradiation of modified ZrO_2_ NPs and probe the effects of O_vS_ relied on photocatalytic properties^26–29^.

## Results and discussion

### Photocatalyst characterization

**Figure 1:**
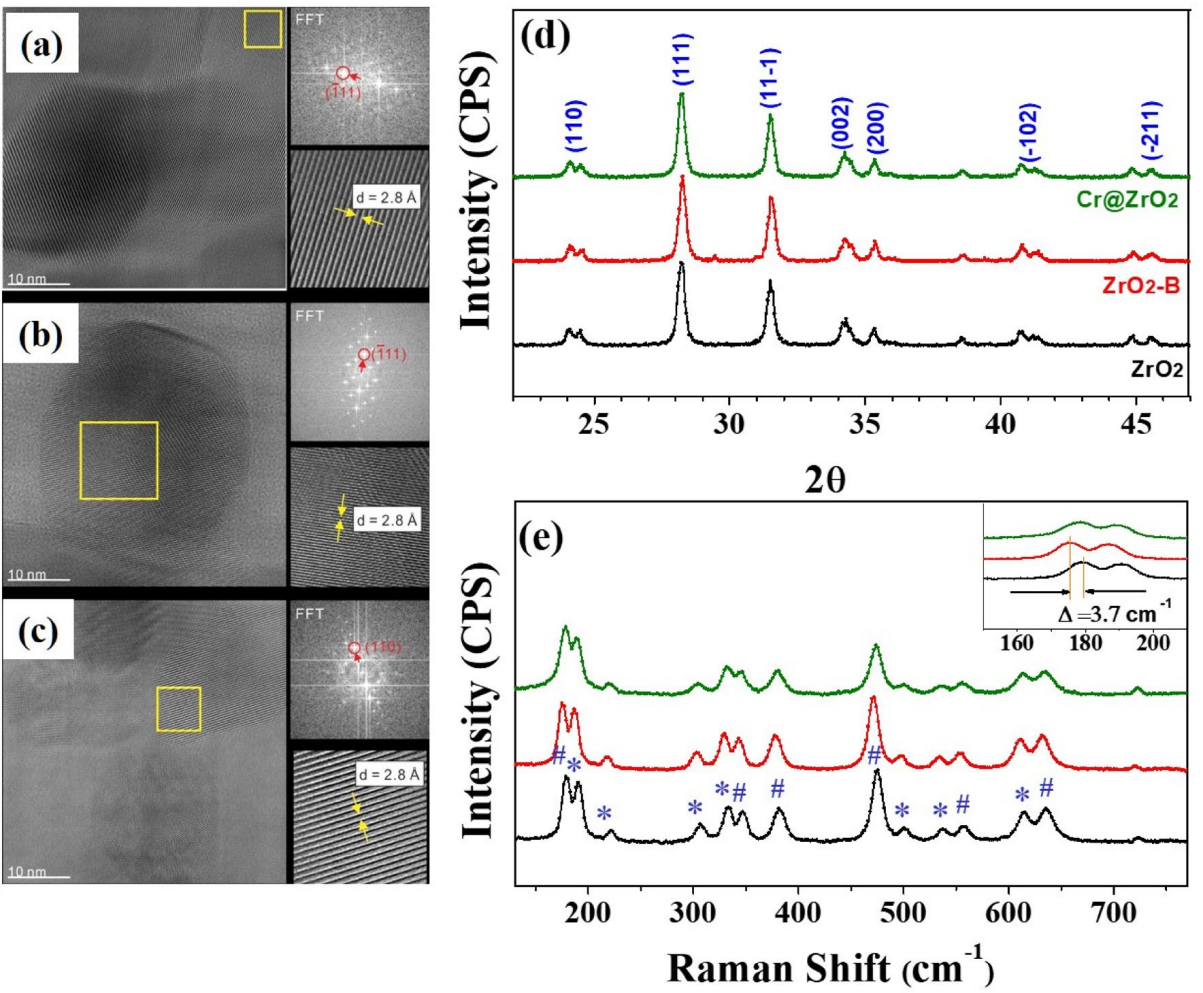
High-resolution TEM images and electron diffraction patterns of (**a**) ZrO_2_ (**b**) ZrO_2_-B, and (**c**) Cr@ZrO_2_ NPs. (**d**) XRD patterns and (**e**) Raman spectra of ZrO_2_, ZrO_2_-B, and Cr@ZrO_2_ NPs. The inset of (**e**) shows the Raman shift of ZrO_2_-B NPs toward the lower energy region. # and * indicate the A_g_ and B_g_ Raman vibrational modes, respectively.

The role of ZrO_2_ NPs surfaces is an important discrepancy that distinguishes photocatalytic activity from catalytic properties. The HR-TEM images of all ZrO_2_ NPs (Fig. 1a–c) featured the typical lattice fringes (111) and (− 111) of monoclinic ZrO_2_ NPs (0.315 and 0.28 nm), which indicated that defect generation did not destroy the crystal structure.

The XRD patterns of all samples (Fig. 1d) exhibited characteristic peaks at 2*θ* = 23.9°, 28.1°, 31.6°, 34.4°, 35.4°, 40.9°, and 45.1°, which corresponded to reflections from the (110), (111), (11−1), (002), (200), (−102), and (−211) planes of monoclinic ZrO_2_ (m-ZrO_2_), respectively^30,31^. Therefore, neither treatment-induced phase transitions. The intensity and width of XRD peaks provide data on crystallite size and structure. Herein, the (111) peak was used to calculate the crystallite sizes of ZrO_2_, ZrO_2_-B, and Cr@ZrO_2_ NPs according to the Scherrer formula as 27.6 ± 0.5, 28.5 ± 0.5, and 26.6 ± 0.5 nm, respectively, which indicated that neither treatment-induced noticeable changes in the particle size distribution^32^.

The Raman spectra of all samples (Fig. 1e) featured characteristic peaks at 178.1 (A_g_ vibrational mode), 190.3 (B_g_ vibrational mode), 221 (B_g_), 309 (B_g_), 332 (B_g_), 345 (A_g_), 380 (A_g_), 478 (A_g_), 503 (B_g_), 539 (B_g_), 558 (A_g_), 613 (B_g_), and 635 cm^−1^ (A_g_)^33,34^. These peaks indicated the presence of m-ZrO_2_ as the dominant phase, in agreement with the results of XRD analysis. We confirm that for base-treated ZrO_2_ NPs (marked as ZrO_2_-B NPs) there was a red-shift of the Raman peaks as shown in the inset of Fig. 1e. This observation clearly supports that the defects were formed because it is relatively weak as the bond length increases. Subsequently, we aimed to determine and compare whether the defect structure formed by base treatment or Cr-ion doping was O_vS_ affecting photocatalytic properties or a simple defect structure. The combined results of HR-TEM, XRD, and Raman spectroscopic analyses revealed that modification did not result in significant structural changes.

### UV light–induced PCD and hydroxyl radical formation

PCD studies were carried out using 4-CP, phenol, and BA as target pollutants to compare the effects of defects on the photocatalytic activity of ZrO_2_ NPs^35,36^. As the large bandgap of ZrO_2_ NPs (5.0 eV) does not allow them to exhibit photocatalytic properties under irradiation with visible light (Fig. S1), the PCD activities of modified ZrO_2_ NPs were assessed using UV light at a wavelength (*λ* ≥ 225 nm).

**Figure 2:**
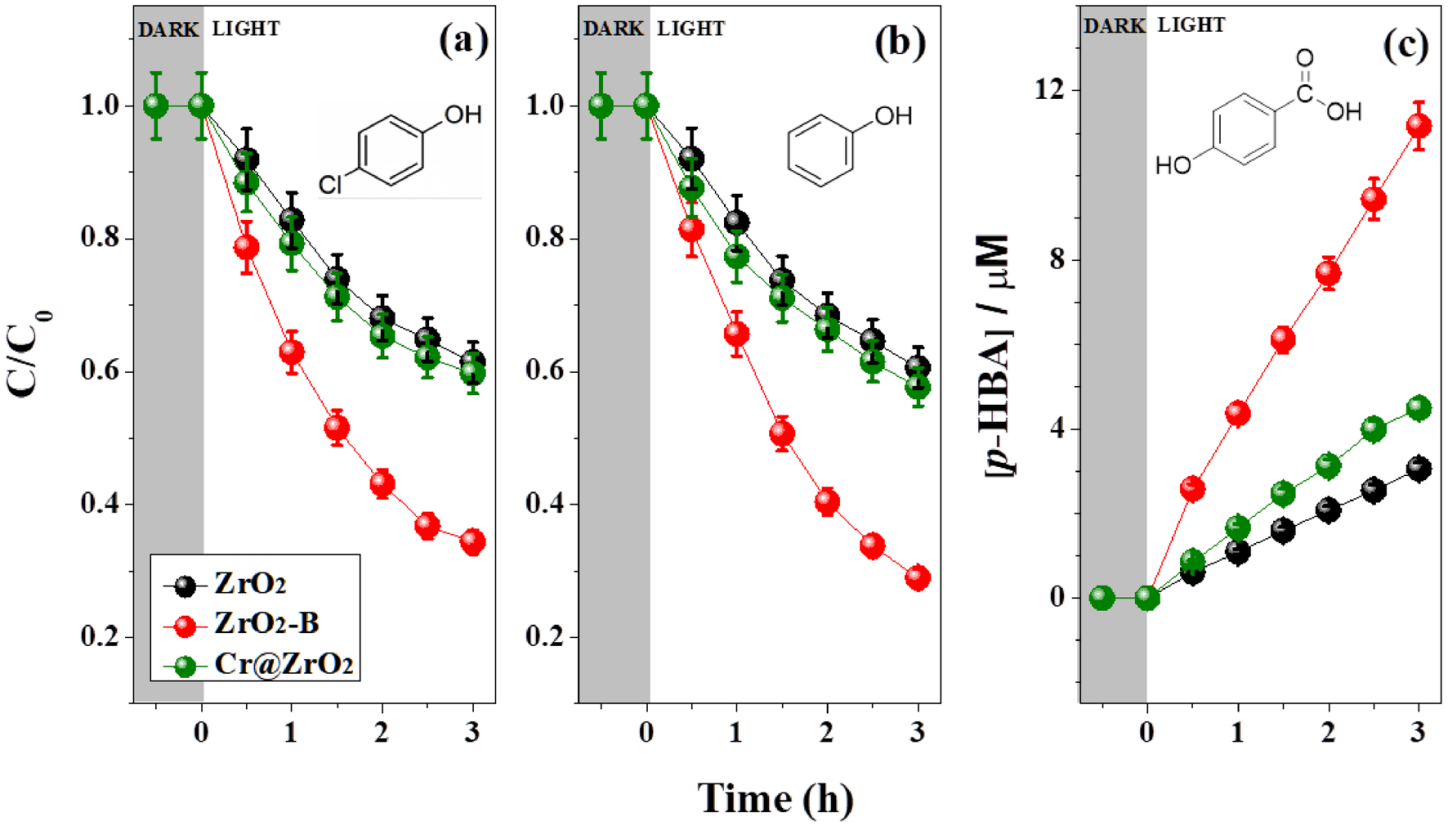
PCD activity of (**a**) 4-CP, (**b**) phenol, and (**c**) BA over ZrO_2_, ZrO_2_-B, or Cr@ZrO_2_ NPs under UV light irradiation (*λ* ≥ 225 nm). The PCD activities extracted from the plots are listed in Table 1.

As shown in Fig. 2 and Table 1, PCD efficiency strongly depended on the modification method. ZrO_2_-B NPs were more efficient at degrading 4-CP and phenol (Fig. 2a,b) than Cr@ZrO_2_ NPs, which indicated that the abundance of O_vS_ in the former catalyst significantly contributed to its enhanced activity. Additionally, by following the production of *p*-HBA from BA, we evaluated the formation of ^**·**^OH over the tested NPs and elucidated the influence of these radicals on the photocatalytic reaction^37^. As shown in Fig. 2c, the ability to produce ^**·**^OH was highest for ZrO_2_-B NPs, which is consistent with the results of the 4-CP and phenol degradation experiments. Hence, base treatment was concluded to be more effective than Cr-ion doping in increasing the amount of O_vS_, which is critical for the improvement of PCD efficiency. Moreover, we could evaluate that Cr_2_O_3_ formed by Cr-ion doping only slightly improved PCD efficiency at wavelengths above 225 nm (see Fig. S1) as we confirm a little effect of PCD activity. Therefore, the doped Cr ions were concluded to act as co-catalysts rather than directly enhancing photocatalytic activity (Fig. S2).

**Table 1:** PCD activities (initial degradation rates (μM min^−1^)) of the evaluated samples after 3 h.

Sample	4-CP	Phenol	BA
Pristine ZrO_2_ NPs	0.53	0.54	3.06
ZrO_2_-B NPs	**0.34**	**0.28**	**11.1**
Cr@ZrO_2_ NPs	0.48	0.47	4.47

The experimental uncertainties are within 5%. The experimental conditions corresponded to [4-CP]_0_ = [phenol]_0_ = 10 µM, [BA]_0_ = 20 µM, [catalyst] = 0.5 g L^−1^, *λ* ≥ 225 nm.

Significant values are in bold.

Catalyst reusability was tested by using recovered ZrO_2_-B NPs to promote the PCD of 4-CP (Fig. S3). As the photocatalytic activity of ZrO_2_-B NPs was maintained for up to five consecutive cycles (15 h in total), the introduced defects were concluded to be stable. In addition, to test the stability of samples after PCD experiments, we measured XPS after the five consecutive photocatalytic cycles. (Fig. S4) After confirming the stability tests, we correlated it with electronic structure using HRXPS and STEM-EELS to explain the high photocatalytic activity of ZrO_2_-B NPs.

### Investigation of defect structures using HRXPS and XAS

HRXPS and XAS were used to analyze the bonding configurations of Zr and O atoms on the surface of NPs and thus evaluate their electronic structures concerning defects.

**Figure 3:**
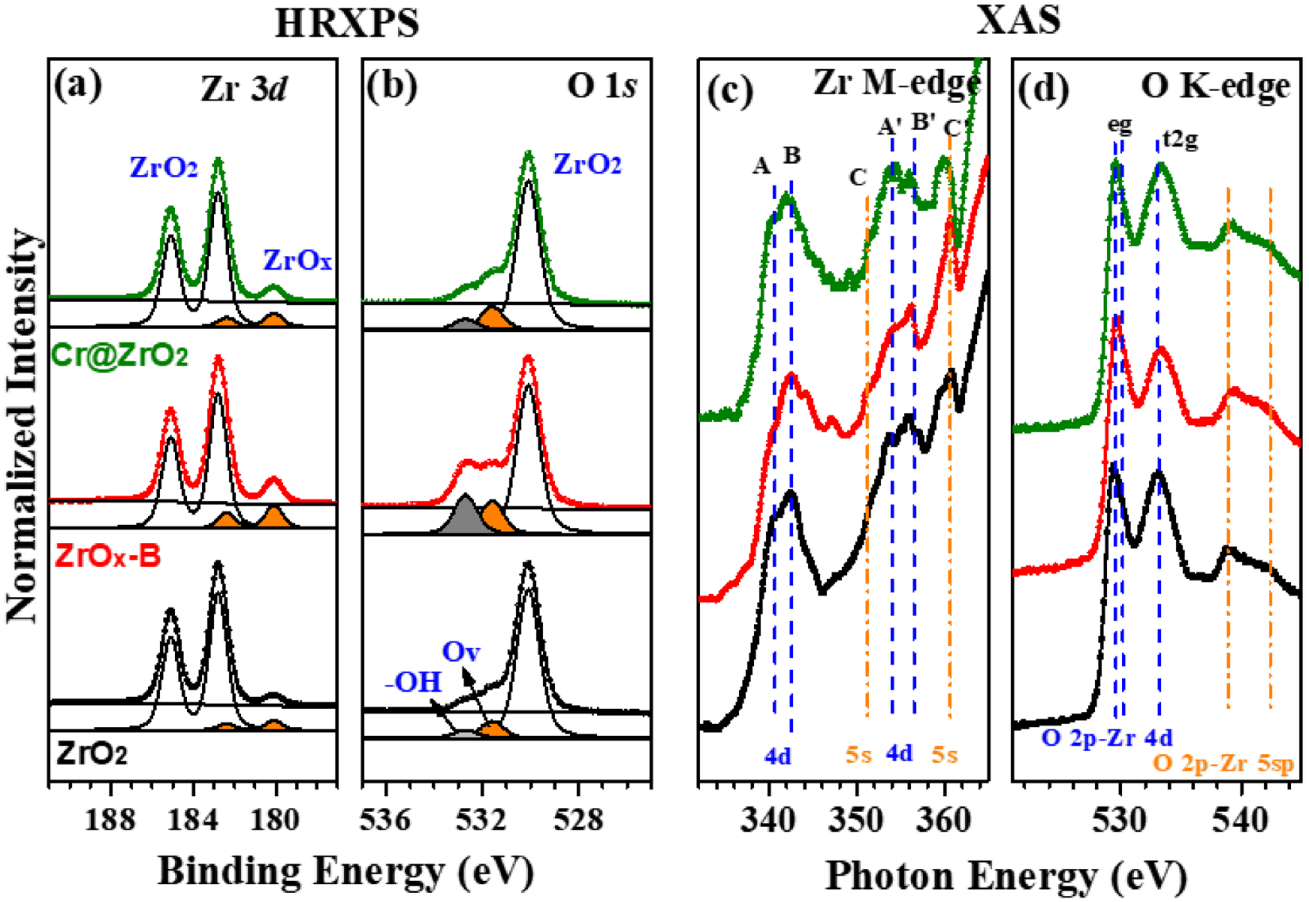
(**a**) Zr 3*d* and (**b**) O 1* s* core-level HR-XP spectra and (**c**) Zr *M*-edge and (**d**) O *K*-edge X-ray absorption spectra of ZrO_2_ (bottom), ZrO_2_-B (middle), and Cr@ZrO_2_ (top) NPs.

The three types of ZrO_2_ NPs exhibited similar core-level spectra that featured two characteristic bonding configurations with differing intensities and thus indicated the varying presence of defect structures. Zr 3*d* core-level spectra (Fig. 3a) featured two peaks at 182.8 and 180.2 eV corresponding to the Zr 3*d*_5/2_ transitions of pristine ZrO_2_ and ZrO_*x*_ with defects, respectively^38,39^. Remarkably, more defects were present in ZrO_2_-B NPs than in Cr@ZrO_2_ NPs, which was in line with the higher PCD activity of the former. After the deconvolution procedure of O 1* s* peaks (Fig. 3b), we exhibit the three distinct components clearly at the binding energy of 530.1 eV (ZrO_2_), 531.6 eV (oxygen vacancy; O_v_), and 532.7 eV (-OH), respectively^40,41^. Focusing on the intensity ratio of –OH and O_v_ peaks, we can identify that the amounts of defect sites of ZrO_2_-B NPs are larger than others, which indicates that treatment with base resulted in surface modification^42,43^. Meanwhile, the intensity change of the ZrO_*x*_ peak is a good indicator for the quantification of defects such as surface hydroxyl-related defects and oxygen vacancies (O_v_s). For ZrO_2_, ZrO_2_-B, and Cr@ZrO_2_ NPs, the ZrO_*x*_/ZrO_2_ (Zr 3*d*) peak intensity ratio equaled ~ 0.08, 0.175, and 0.097, respectively. The intensity ratio difference between the samples prepared by the two modification methods confirmed that base treatment is more effective at forming O_vS_ than Cr-ion doping.

Figure 3c,d show the X-ray absorption spectra of ZrO_2_ NPs. Based on dipole selection rules, signals in the Zr M-edge spectra (Fig. 3c) were assigned to transitions between the *M*_2,3_ *p*-core states of Zr atoms and the conduction band states derived from the 4*d* (A and B and A’ and B’) and the 5* s* (C and C’) atomic states of Zr^44,45^. As in the case of XPS analysis, the spectrum of ZrO_2_-B NPs was markedly different from those of the other two samples, featuring a strongly attenuated peak A, which may indicate an increased defect content. A similar trend was observed for O *K*-edge spectra (Fig. 3d), in which case the decrease in the intensity of the t_2g_ peak observed for ZrO_2_-B NPs was explained by a change in defect structure. Thus, the intensities of defect-attributable peaks (ZrO_*x*_) in the spectra of the base-treated sample exceeded those in the spectra of the Cr-ion-doped sample. Consequently, the apparent difference between the X-ray absorption spectrum of ZrO_2_-B NPs and those of the other two samples was attributed to remarkable changes in defect sites due to base treatment, which agreed with the results of HRXPS analysis. The enhanced photocatalytic activity of the ZrO_2_-B NPs shows that OH- and O_vS_ formed around the defect structure can form a relatively large amount of ^**·**^OH affecting the photocatalytic reaction of the ZrO_2_ NPs^46,47^. Moreover, to verify the oxidation states of the doped Cr ions in Cr@ZrO_2_ NPs, we investigate the electronic states of Cr by using HRXPS and XAS as shown in Fig. S5.

### STEM-EELS and in situ XPS during UV irradiation

As the difference in the defect site ratio determined using HRXPS represented the average over many NPs, further analysis was required to precisely compare the number of defect sites for single NPs. Thus, to obtain site-specific defects data for the surface and core of a single NP, we used high-resolution STEM-EELS to detect changes in the spectral profile of the O *K*-edge, including those in the defect-induced peak (surface hydroxyl-related defects or O_v_s)^48,49^.

**Figure 4:**
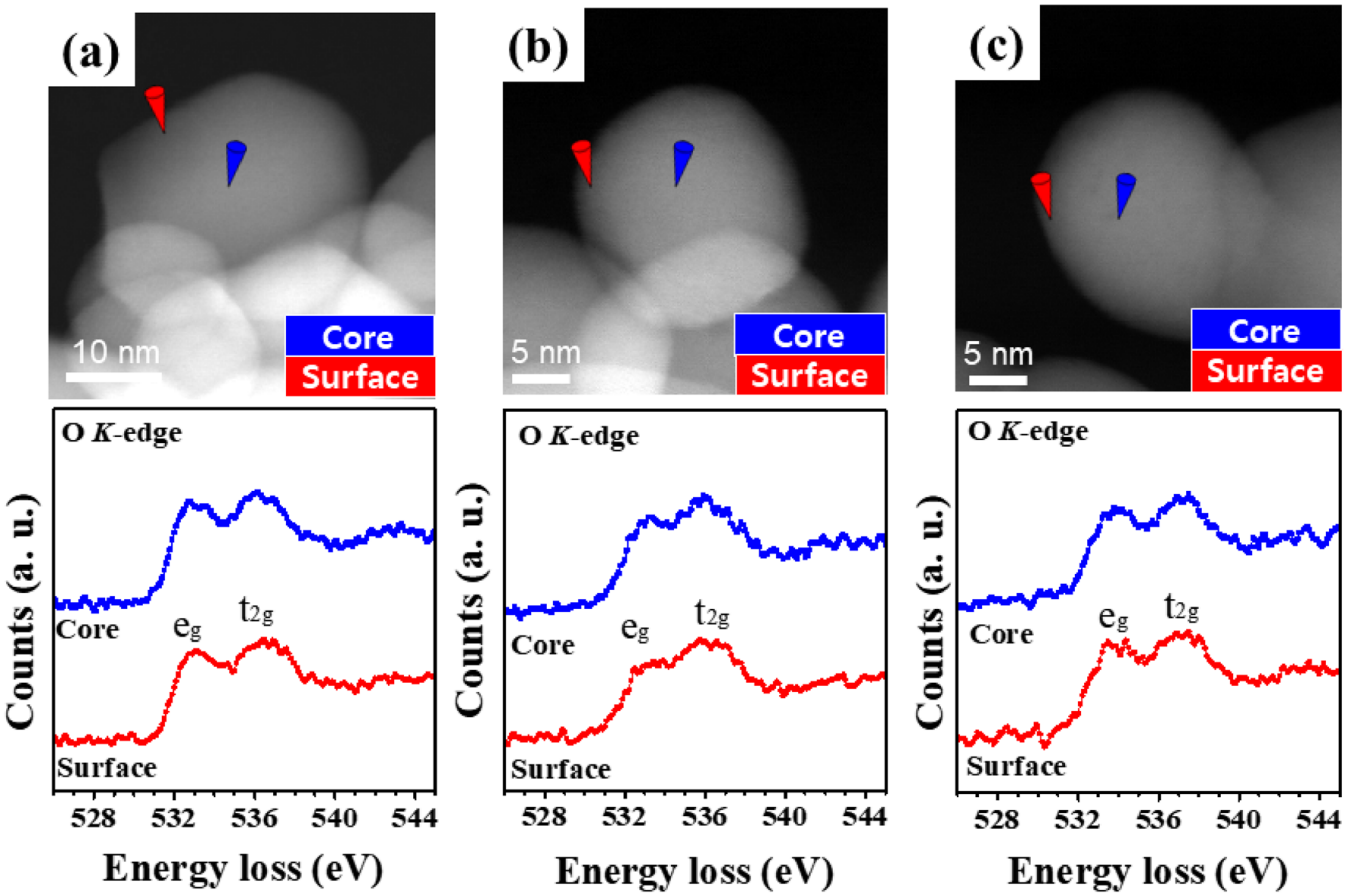
(**a**–**c**) STEM–EELS images and O *K*-edge ELNES spectra of (**a**) ZrO_2_, (**b**) ZrO_2_-B, and (**c**) Cr@ZrO_2_ NPs. Red and blue cones indicate the probing sites at the surface and the core of each NP, respectively.

Figure 4 shows the O *K*-edge energy-loss near-edge structure (ELNES) spectra obtained for the surface and core sites of each NP. In the energy loss range of 525–545 eV, two peaks corresponding to e_g_ (~ 533.2 eV) and t_2g_ (~ 536.4 eV) were observed, corresponding to the hybridization of O 2*p* and Zr 4*d* states, respectively. The e_g_/t_2g_ peak intensity ratio changes with the amounts of O_vS_ because of the formation of deep donor states due to oxygen deficiency and is, therefore, a sensitive indicator of the relative concentration of O_vS_ within the investigated spatial region^50^. The O-*K* ELNES spectra of a single pristine ZrO_2_ NP (Fig. 4a) exhibited the features typical of ZrO_2_ NPs with no changes in the above intensity ratio (which equaled 0.89 ± 0.03 and 0.96 ± 0.02 for the surface and the core, respectively) or the spectral profile^51^. A prominent change was observed for ZrO_2_-B NPs (e_g_/t_2g_ = 0.78 ± 0.04 (surface) and 0.92 ± 0.03 (core)), suggesting a significant alteration of electronic structure and indicating that base treatment significantly affected the amounts of O_vS_ and thus increased PCD activity. Conversely, no significant difference in the amounts of O_vS_ was observed between Cr-doped (e_g_/t_2g_ = 0.87 ± 0.05 (surface) and 0.95 ± 0.02 (core)) and pristine samples. This confirms that base treatment had a significantly larger effect on O_vS_ than Cr-ion doping, which is directly related to the photocatalytic activity.

Furthermore, the distribution of O_vS_ within a single NP can be evaluated by comparing the e_g_/t_2g_ peak intensity ratios of the surface and core regions of each particle. Herein, the ZrO_2_-B NPs exhibited an e_g_/t_2g_ peak intensity ratio ⁓11.5% lower than that of Cr@ZrO_2_ NPs in all particle regions.

As shown in Fig. 2, ZrO_2_-B NPs exhibited higher PCD activity than other samples. HRXPS (Fig. 3) also allowed us to distinguish the hydroxyl-induced oxygen vacancy of ZrO_2_-B NPs from the defect structures of Cr@ZrO_2_ NPs. To investigate differences in the PCD activities of the three samples due to defect formation, we used XPS under irradiation with UV light of the same wavelength (*λ* ≥ 225 nm) as that used for the PCD reaction^28,52^.

**Figure 5:**
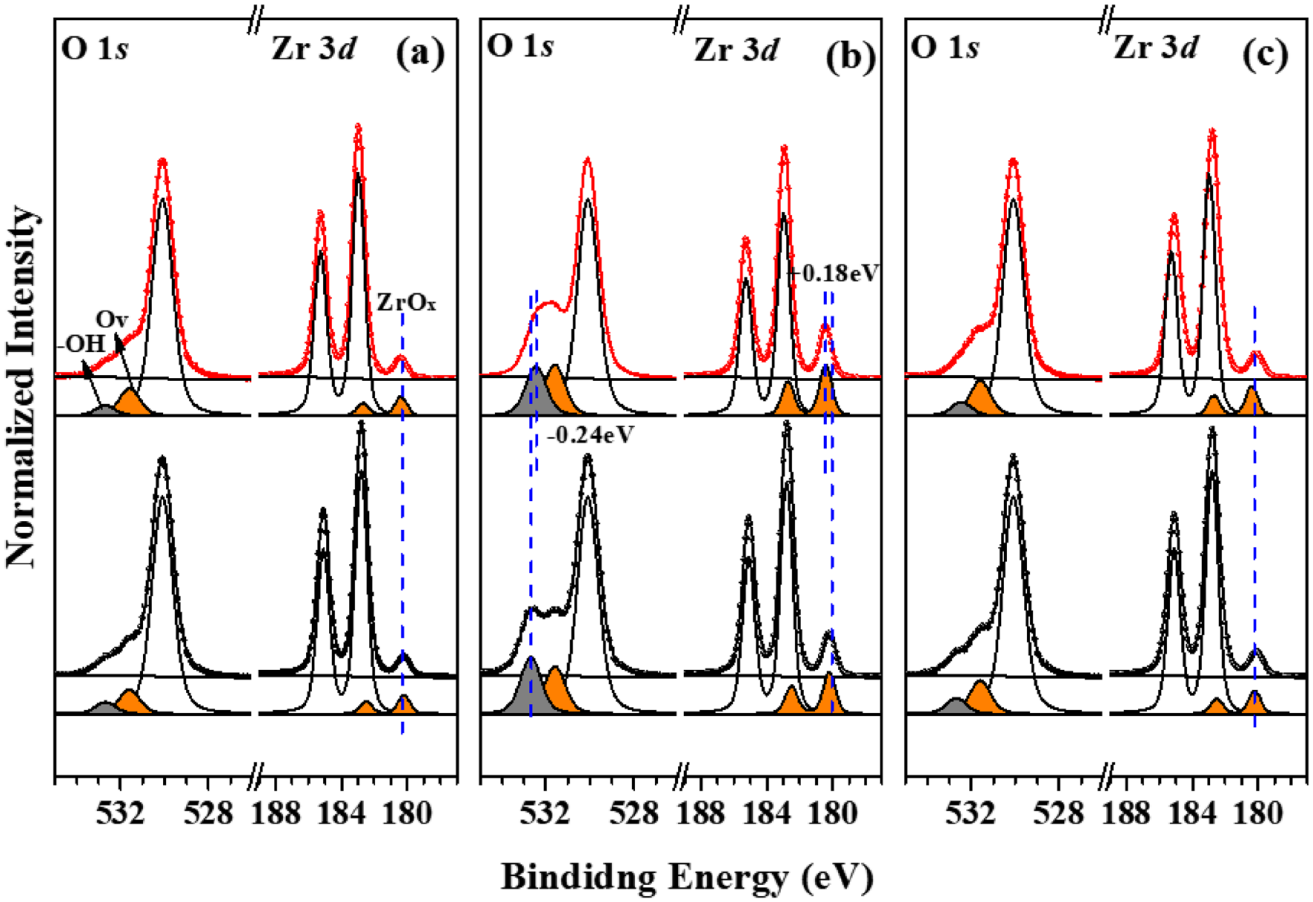
In situ X-ray photoelectron spectra of (**a**) ZrO_2_, (**b**) ZrO_2_-B, and (**c**) Cr@ZrO_2_ NPs recorded with (red) and without (black) UV light irradiation (*λ* ≥ 225 nm).

Figure 5 shows changes in the in situ X-ray photoelectron spectra (Zr 3*d* and O 1* s*) of the three samples recorded with and without UV irradiation (*λ* ≥ 225 nm). As expected, a large change was observed for ZrO_2_-B NPs (Fig. 5b). Regarding Zr 3*d* spectra, no shift of the ZrO_2_ peak was observed for any sample upon light on/off. However, the ZrO_*x*_ peak due to photocatalytic-related O_vS_, exhibited a large shift, particularly in the case of ZrO_2_-B (0.18 eV). To explain this behavior, we focused on the intensity change of the ZrO_*x*_ peak in ZrO_2_-B NPs. Upon irradiation with additional 225-nm light, the intensity of the ZrO_*x*_ peak of ZrO_2_-B NPs increased by ~ 13.5%, whereas no such increase was meaningfully observed for other samples. This finding is consistent with the results of PCD activity evaluation, according to which only ZrO_2_-B NPs exhibit enhanced photocatalytic activity. Therefore, the increased O_vS_ of ZrO_2_-B NPs underwent a large change due to UV light irradiation, which explains the difference in the results of PCD activity evaluation.

The same trend was observed for O 1*s* core-level spectra. Specifically, in the spectrum of ZrO_2_-B NPs, the Zr–OH peak corresponding to surface hydroxyl-related defects exhibited a shift of ~ 0.24 eV and an intensity decrease of 13.0%, and O_v_ peaks show an increase of approximately 13.0% at the same time, which was attributed to the enhanced photocatalytic properties of this sample. Therefore, the presence of many -OH groups on the surface of ZrO_2_-B NPs was indicative of a more O_vS_ structure, which is closely related to PCD activity. Conversely, the intensity changes of the ZrO_*x*_ peak in the X-ray photoelectron spectra of ZrO_2_ and Cr@ZrO_2_ NPs were not vivid and were almost equivalent to those observed for the ZrO_2_ peak during UV light irradiation, i.e., UV light irradiation induced only a small change in the oxygen-deficient structure. Therefore, unlike that of ZrO_2_-B NPs, the photocatalytic activity of these two samples did not markedly improve. The results of in situ XPS measurements precisely confirmed the existence of many –OH groups can be an indicator of the formation of abundant O_vS_ upon irradiation and demonstrated that the extent of this transfer was enhanced in ZrO_2_-B NPs. The peak shift values of ZrO_x_ in Zr 3*d* and –OH on O 1*s* core-level spectra of the three samples are listed in Table 2.

**Table 2:** Core-level shift of Zr 3d and O 1s peak during UV irradiation (*λ* ≥ 225 nm).

Sample	Zr 3*d* (eV) ZrO_x_ peak	O 1*s* (eV) –OH peak
Pristine ZrO_2_ NPs	0.05 ± 0.02	0.06 ± 0.01
ZrO_2_-B NPs	**0.18** ± **0.05**	**0.24** ± **0.08**
Cr@ZrO_2_ NPs	0.05 ± 0.02	0.05 ± 0.01

Significant values are in bold.

Notably, although defects were observed in both ZrO_2_-B and Cr@ZrO_2_ NPs, they exhibited markedly different photocatalytic properties, which was correlated with the presence/absence of ^**·**^OH on the NP surface, which originated from O_vS_. Among these two catalysts, ZrO_2_-B NPs featured –OH peak larger than others in their high-resolution X-ray photoelectron spectrum, thus forming relatively larger amounts of O_vS_. Even for similar oxygen-deficient structures, photocatalytic activity considerably increased in the presence of many -OH groups. To investigate the improvement of the photocatalytic properties of ZrO_2_-B NPs with many O_vS_, we additionally conducted an experiment on free radical trapping using radical scavenger (5,5-Dimethyl-1-pyrroline N-oxide; DMPO). As expected, when the radical scavenger (DMPO; 20 µM) was added together with 4-CP (10 µM) to cause the PCD reaction, it was confirmed that DMPO reacted preferentially with the ^**·**^OH radical generated by the photoreaction and that it interrupt the PCD reaction of the ZrO_2_–B NPs. (Fig. S6).

As is evidently in Fig. S2, it confirms that the PCD extent of 4-CP and thiophenol depended on the concentration of doping Cr ions in Cr@ZrO_2_ NPs, which indicates that Cr_2_O_3_ facilitates oxidation rather than acts as a photocatalyst.

Lastly, we found that the base treated ZrO_2_ shows the enhanced PCD activity due to many OH- at the surface of ZrO_2_ NPs. To clarify this effect, we also did pH dependent PCD test while changing the pH solution from 7.0 to 13.0. As expected, we confirmed the larger the pH value, the greater the change was due to the formation of many OH- ions under basic conditions. However, the change in PCD activity according to the change in the pH was not shown proportionally though as shown in Fig. S7.

**Scheme 1:**
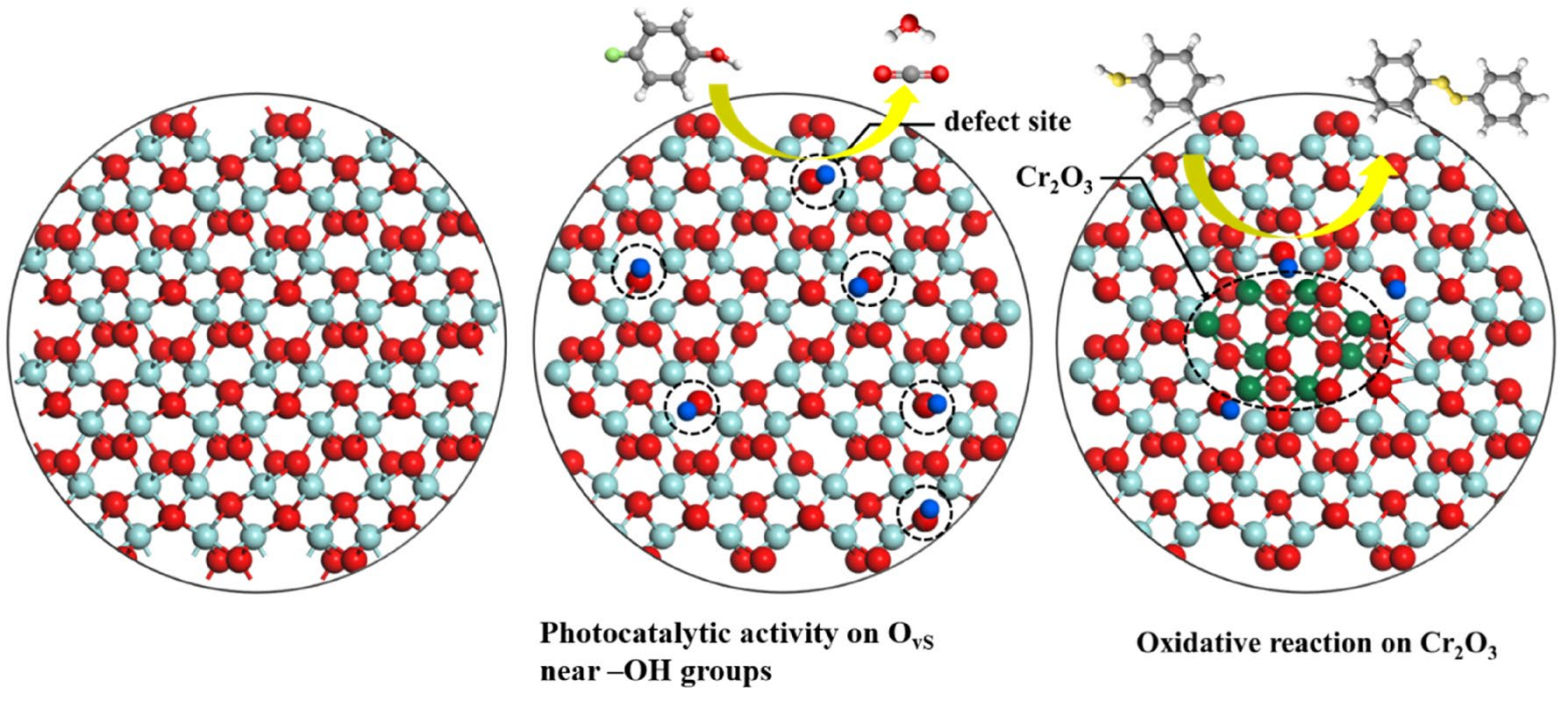
The behavior of O_v_ sites on ZrO_2_ NPs modified using two different methods during PCD.

Consequently, compared to Cr-ion doping, base treatment was better at improving the photocatalytic properties of ZrO_2_ NPs, increasing the number of ^**·**^OH near the -OH groups (Scheme 1).

## Conclusions

Modified ZrO_2_ NPs were prepared to use base treatment and Cr-ion doping, and the former treatment was shown to have a larger effect on photocatalytic properties than the latter. Mesoscale HRXPS, XAS, and atomic-scale STEM-EELS measurements showed that the enhanced PCD activity of the base-treated sample was due to an increase in the content of defect sites without a marked alteration of atomic structure. The rates of the ZrO_2_ NPs-catalysed PCD of organic pollutants were strongly affected by modification type, as it influenced the defect sites and, hence, oxygen-storage properties and the adsorption and reduction of dissolved O_2_. Using in situ XPS, we demonstrated that defect sites (O_v_) formed in the presence of many –OH groups at the ZrO_2_ NP surfaces directly affect photocatalytic activity. Thus, our study provides a novel approach for the development of highly stable defect-engineered photocatalysts.

## Material and methods

### Synthesis of pristine ZrO_2_ NPs

A solution of zirconium isopropoxide (12 g) in ethanol (80 mL) was added to distilled deionized water (DDW; 500 mL) over 1 h upon gentle stirring, and the reaction mixture was heated in an autoclave at 220 °C for 10 h. After cooling to 25 °C, the produced ZrO_2_ NPs were selectively precipitated, dried at 90 °C for 48 h, and used for the synthesis of base-treated ZrO_2_ NPs (ZrO_2_-B NPs) or Cr-doped ZrO_2_ NPs (Cr@ZrO_2_ NPs)^53,54^.

### Synthesis of ZrO_2_-B NPs

An aqueous solution with a KOH-adjusted pH of 13 was allowed to stand for 2 h and then supplemented with pristine ZrO_2_ NPs (3.6 g) upon stirring. The gel solution obtained after ~ 30 min was transferred to an autoclave and heated at 220 °C for 8 h in a convection oven. The reaction mixture was cooled to 25 °C, and the produced ZrO_2_-B NPs were precipitated, washed with DDW, and dried at 90 °C for 48 h.

### Synthesis of Cr@ZrO_2_ NPs

Pristine ZrO_2_ NPs (3.0 g) were added to a solution of Cr(NO_3_)_3_·9H_2_O (0.612 g) in DDW (100 mL). After 30-min stirring at 90 °C, the heterogeneous mixture was transferred to an autoclave and heated at 220 °C for 8 h in a convection oven. The obtained Cr@ZrO_2_ NPs were precipitated, washed with DDW, and dried at 90 °C for 48 h.

### Evaluation of photocatalytic performance

The catalyst of choice (0.015 g) was dispersed in distilled water (30 mL). The resulting suspension was stirred for 30 min to allow the adsorption of 4-CP, phenol, or BA on the NPs to reach equilibrium and then irradiated with a 300-W Xe arc lamp (Oriel Lighting, Darra, QLD, Australia) equipped with a cut-off filter (*λ* ≥ 225 nm). Aliquots were intermittently withdrawn using a 1-mL syringe and filtered through a 0.45-μm polytetrafluoroethylene filter (Millipore Sigma, Burlington, MA, USA) to remove the suspended ZrO_2_ NPs. The filtrates were analyzed by high-performance liquid chromatography (LC-20AD Pump, Shimadzu, Kyoto, Japan) to quantify the residual 4-CP, phenol, or BA and thus determine photocatalytic activity.

### Instrumentation

The phase composition was probed by X-ray diffraction (XRD; M18XHF, MAC Science Co., Yokohama, Japan), while surface functional groups were probed by Raman spectroscopy (Labram ARAMIS instrument with an Ar^+^-ion continuous-wave (514.5 nm) laser, Horiba Ltd., Kyoto, Japan). Schottky field-emission STEM measurements were performed at 200 kV using an instrument equipped with a probe-forming spherical aberration (*C*_s_) corrector (JEM-2100F, JEOL Ltd., Tokyo, Japan). Bright-field high-resolution transmission electron microscopy (HR-TEM) images were captured by a charge-coupled device camera (OneView, Gatan, Inc., Pleasanton, CA, USA) at full 4 k × 4 k resolution using an acquisition time of 16 s. Chemical composition was probed by STEM coupled with energy-dispersive X-ray spectroscopy (STEM-EDS; JED-2300T, JEOL Ltd., Tokyo, Japan), while O *K*-edge STEM-EELS measurements (GIF Quantum ER, Gatan, Inc., Pleasanton, CA, USA) were carried out at an energy resolution of 0.8 eV and a dispersion of 0.1 eV/pixel for an exposure time of 1.0 s. Electronic structures were probed by HRXPS and XAS at the 10A2 beamline of the Pohang Accelerator Laboratory. During in situ XPS measurements, which were performed at the above beamline, a low-power 225 nm light-emitting diode (Solis High-Power LED, Thorlabs, Inc., Newton, NJ, USA) was placed at ~ 25 cm from the samples to investigate electron density changes under light irradiation.

## Supplementary Information

Supplementary Information.

## Data Availability

The datasets generated during and/or analyzed during the current study are available from the corresponding author on reasonable request.
