# Supplementary material for: Verifying the relationships of defect site and enhanced photocatalytic properties of modified ZrO2 nanoparticles evaluated by in-situ spectroscopy and STEM-EELS
Source: Sci Rep. 2022 Jul 4;12:11295. doi: 10.1038/s41598-022-15557-0 (PMC9253032; doi:10.1038/s41598-022-15557-0)
Supplement: Supplementary file 1 — Supplementary Information. [file 41598_2022_15557_MOESM1_ESM.docx]

Electronic Supplementary Information

**Verifying the Relationships of Defect Site and Enhanced Photocatalytic Properties of Modified ZrO_2_ Nanoparticles Evaluated by In situ Spectroscopy and STEM-EELS**

Hyun Sung Kim,^a,†^ Ye-Jin Kim,^b,†^ Ye Rim Son,^a,e^ Vy Ngoc Pham,^c^ Ki-jeong Kim,^d^ Chang Woo Kim,^e^ Young-Sang Youn,^f^ Oh-Hoon Kwon,^b,^* and Hangil Lee^c,^*

^a^Department of Chemistry, Pukyong National University, Busan 48513, Republic of Korea

^b^Department of Chemistry, Ulsan National Institute of Science and Technology, Ulsan 44919, Republic of Korea

^c^Department of Chemistry, Sookmyung Women’s University, Seoul 04310, Republic of Korea

^d^Beamline Research Division, Pohang Accelerator Laboratory, Pohang 37673, Republic of Korea

^e^Department of Smart and Green Technology Engineering, Pukyong National University, Busan 48513, Republic of Korea

^f^Department of Chemistry, Yeungnam University, Daehak-ro 280, Gyeongsan, Gyeongbuk 38541, Republic of Korea

**AUTHOR INFORMATION**

^†^ These authors contributed equally to this manuscript.

*Corresponding Author;

E-mail addresses: easyscan@sookmyung.ac.kr (H. L.), phone: +82-2-710-9409 (H.L.)

E-mail addresses: ohkwon@unist.ac.kr (O.-H. K.), +82-52-217-5653 (O.-H. K.)

**S1. UV-Vis spectra**


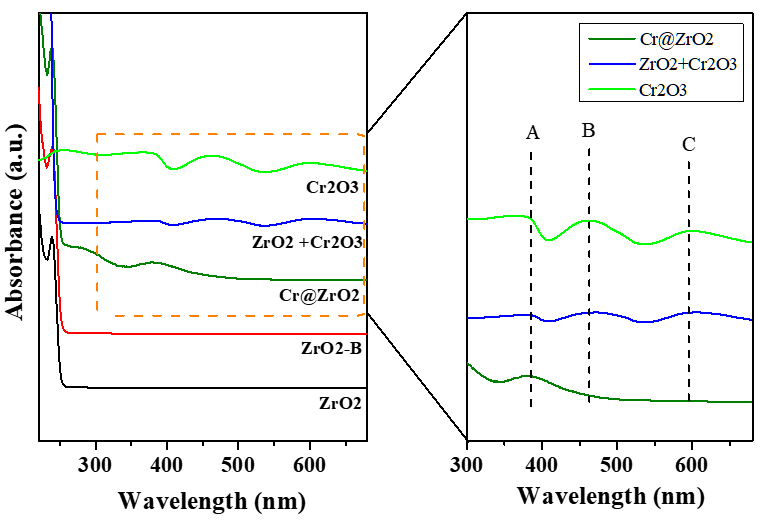


**Figure S1**. (Left panel) UV-Vis spectra of ZrO_2_, ZrO_2_-B, Cr@ZrO_2_, physically mixed ZrO_2_+Cr_2_O_3_, and Cr_2_O_3,_ and (right panel) zoom-in spectra of Cr@ZrO_2_, physically mixed ZrO_2_+Cr_2_O_3_, and Cr_2_O_3_ NPs.

As shown in Fig. S1, all three samples (ZrO_2_, ZrO_2_-B, and Cr@ZrO_2_ NPs) show a bandgap of approximately 4.97 eV. To clarify the three absorption peaks, we additionally measured Cr_2_O_3_ and physically mixed ZrO_2_ and Cr_2_O_3_. The peaks appear at 386 (A), 462 (B), and 595 (C) nm wavelengths in the spectrum of Cr_2_O_3_ or physically mixed Cr_2_O_3_+ZrO_2_, which are typical spectra of Cr_2_O_3_. In the case of Cr@ZrO_2_ NPs, peaks appear at 386 nm. It is noted that a small amount of Cr^3+^ ions are doped into ZrO_2_ NPs. Therefore, it can be confirmed that the charge state of Cr is Cr^3+^ electronic state in Cr@ZrO_2_ NPs.

**S2. Oxidative and photocatalytic measurements**


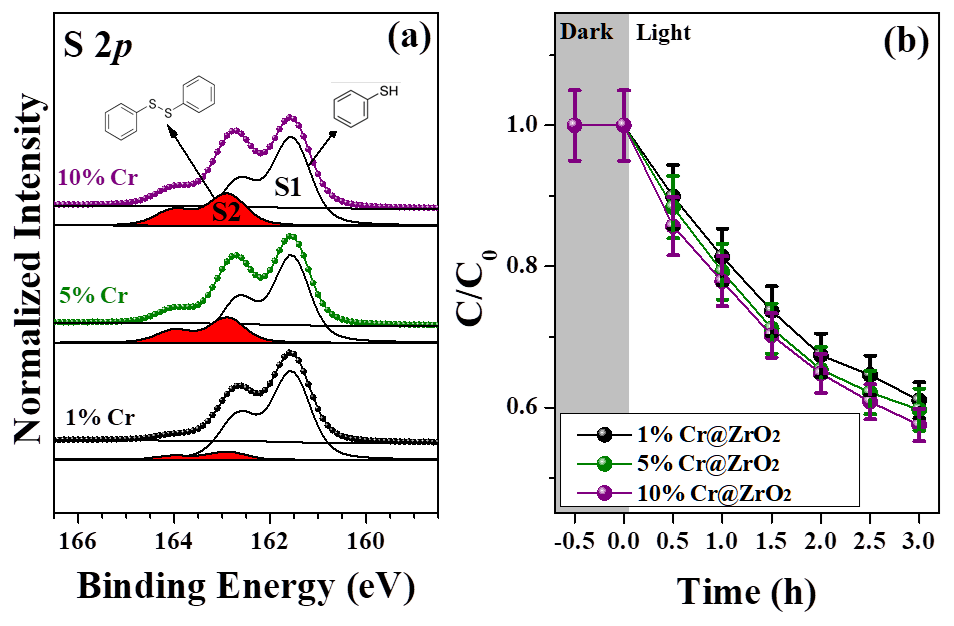


**Figure S2**. (a) S 2*p* core-level spectra of oxidation reaction of thiophenol and (b) 4-CP PCD of Cr@ZrO_2_ NPs depending on the amount of Cr ions under UV light irradiation (*λ* ≥ 225 nm).

The PCD and HRXPS analyses suggested that Cr_2_O_3_ acted not as a photocatalyst but as a catalyst. To confirm this claim, we performed the oxidation reaction of thiophenol and PCD of 4-CP depending on the Cr ions concentration, as shown in Fig. S2. It is possible to confirm the role of the optimized catalyst Cr_2_O_3_ as we tracked the changes in the oxidation reaction by Cr_2_O_3_. The oxidation reaction of thiophenol by the Cr@ZrO_2_ NPs shown in Fig. S3a clearly increased the degree of the oxidation reaction depending on the amount of doped Cr ions. The thiophenol molecule (marked as S1) is further oxidized to a disulfide-type molecule (marked as S2) on the surfaces of the Cr@ZrO_2_ NPs according to the Cr ion doping amount [S1, S2]. On the other hand, in the case of the PCD reaction for the 4-CP molecule shown in Fig. S2b, the photocatalytic efficiency of the Cr ion is not significantly changed, even though the amount of Cr ions increases. Considering the error range, there is almost no increase in the effect. The comparison of the oxidation and PCD reactions by changing the amount of Cr ions (Cr@ZrO_2_ NPs), as described above, shows that the Cr@ZrO_2_ NPs favored the role of a catalyst rather than a photocatalyst.

**S3**. **Repeated cycles of 4-CP degradation test**


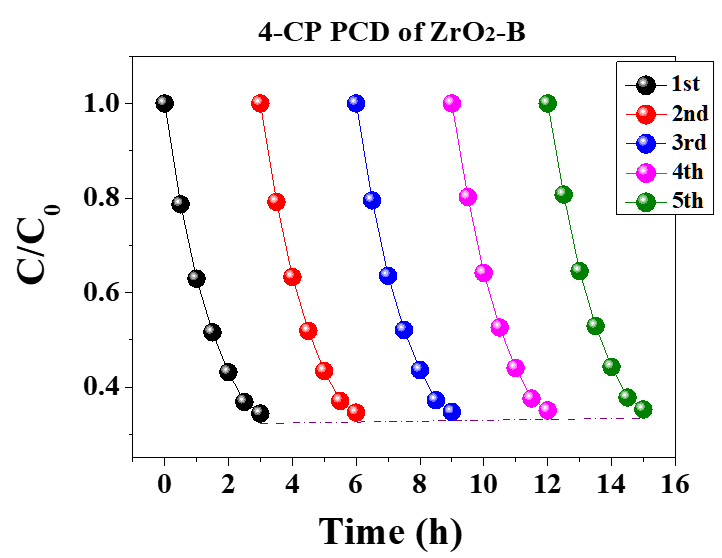


**Figure S3.** Repeated cycles of 4-CP degradation with ZrO_2_-B NPs under UV light irradiation (*λ* ≥ 225 nm). The experimental conditions were as follows: [ZrO_2_-B] = 0.5 g/L, [4-CP]_0_ = 100 µM

**S4. Stability test of the samples using XPS after the five consecutive photocatalytic cycles.**


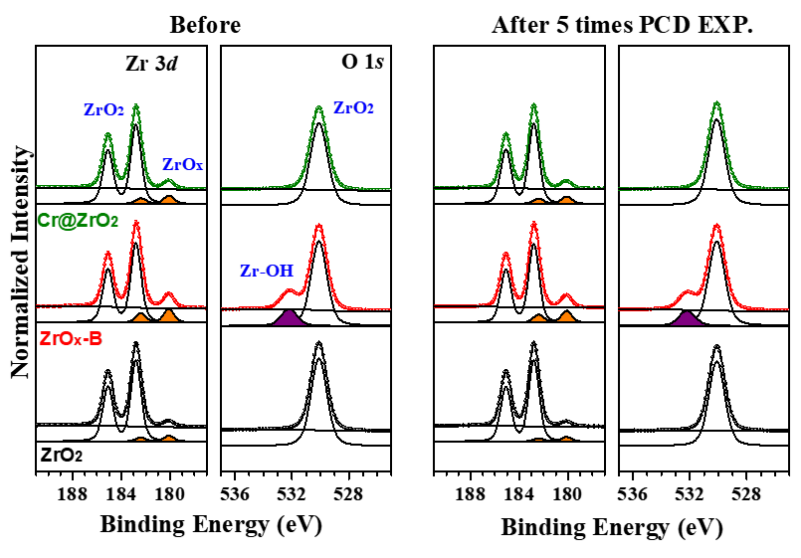


**Figure S4.** XPS spectra of Zr 3d and O 1s core levels for three different samples were measured before and after five repetitions of the PCD experiment.

For the Zr 3d peaks of ZrO_x_ of all three samples corresponding to the defect structures, the intensity did not change even after repeating PCD experiments five times. In addition, there was no significant change in the O 1*s* peaks. From these results, we confirm that the defect structures of all samples are intact after the photocatalytic reactions.

**S5. Confirming electronic states of the doped Cr ions**


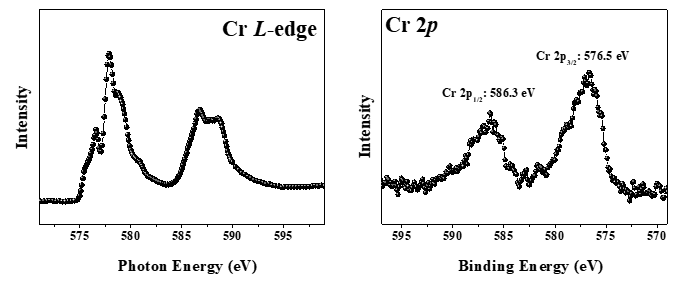


**Figure S5**. (left) XAS spectra of Cr *L*-edge and (right) XPS spectrum of Cr 2*p* core level.

We measured XPS and XAS for Cr oxide formed at the surface of ZrO_2_ NPs to investigate the oxidation states of the doped Cr ions. As shown in Fig. S5, both spectra show a typical electronic structure for Cr_2_O_3_. The peaks of XPS spectra of Cr 2*p* core-level spectrum at 576.5 and 586.3 eV should be assigned to Cr^3+^ 2p_3/2_ and Cr^3+^ 2p_1/2_. Therefore, the oxidation number of Cr is 3+. Also, the Cr *L*-edge spectrum obtained by XAS measurement was shown to be typical Cr_2_O_3_. From the two methods (XPS and XAS), it is can be confirmed that the oxidation number of Cr is 3+ charge state. It means that both of Cr in the ZrO_2_ lattice and in Cr_2_O_3_ lumps are Cr^3+^ valence states. However, since the amount of the doped Cr^3+^ state inside ZrO_2_ lattice is too low, it is expected that the defect structure that affects the photocatalyst does not form much.

**S6. Free radical trapping experiment**


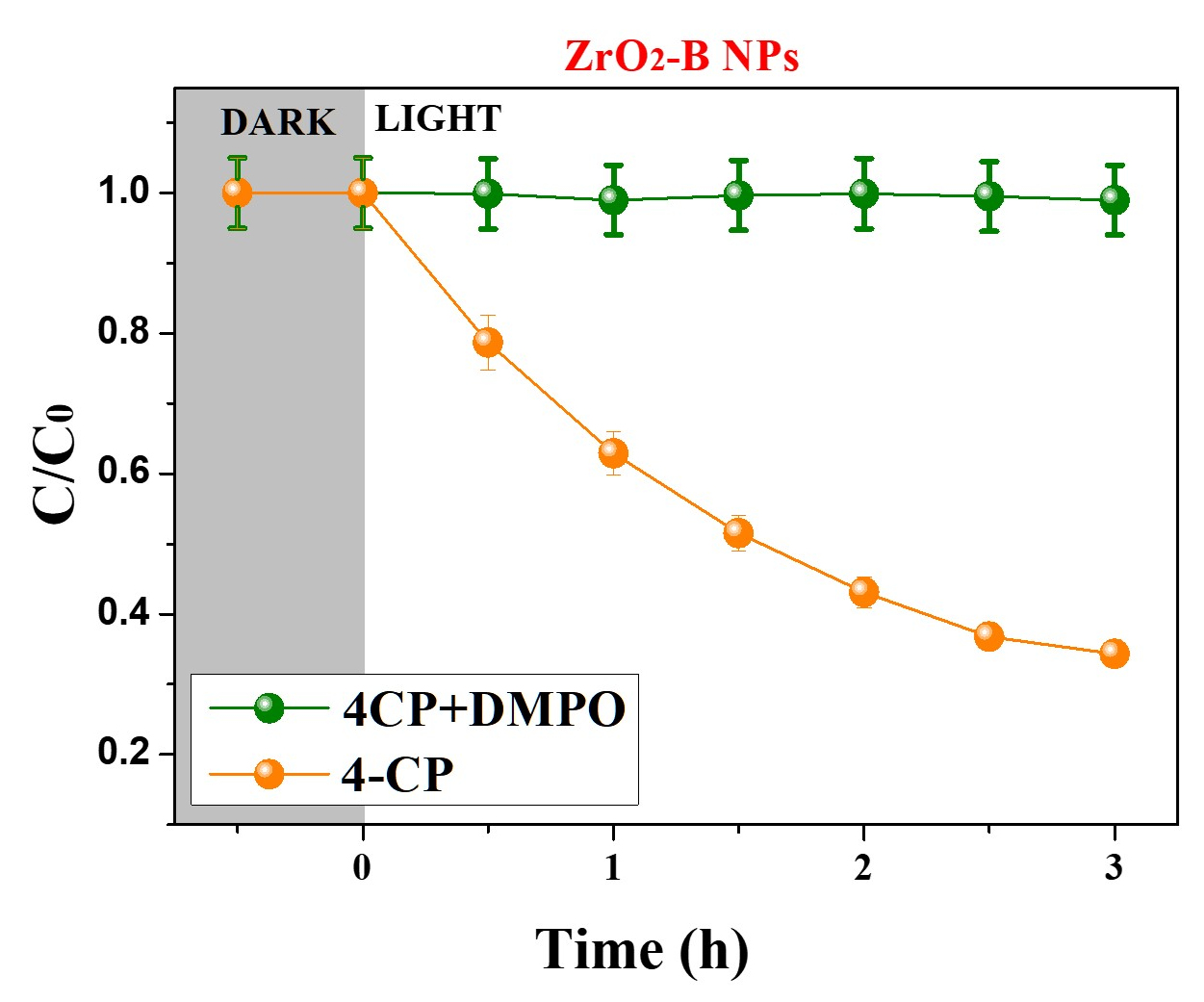


**Figure S6**. PCD activity of ZrO_2_–B NPs using 4-CP with or without DMPO (radical scavenger) under UV light irradiation (*λ* ≥ 225 nm).

**S7. pH dependent PCD activity of ZrO_2_-B NPs**

**
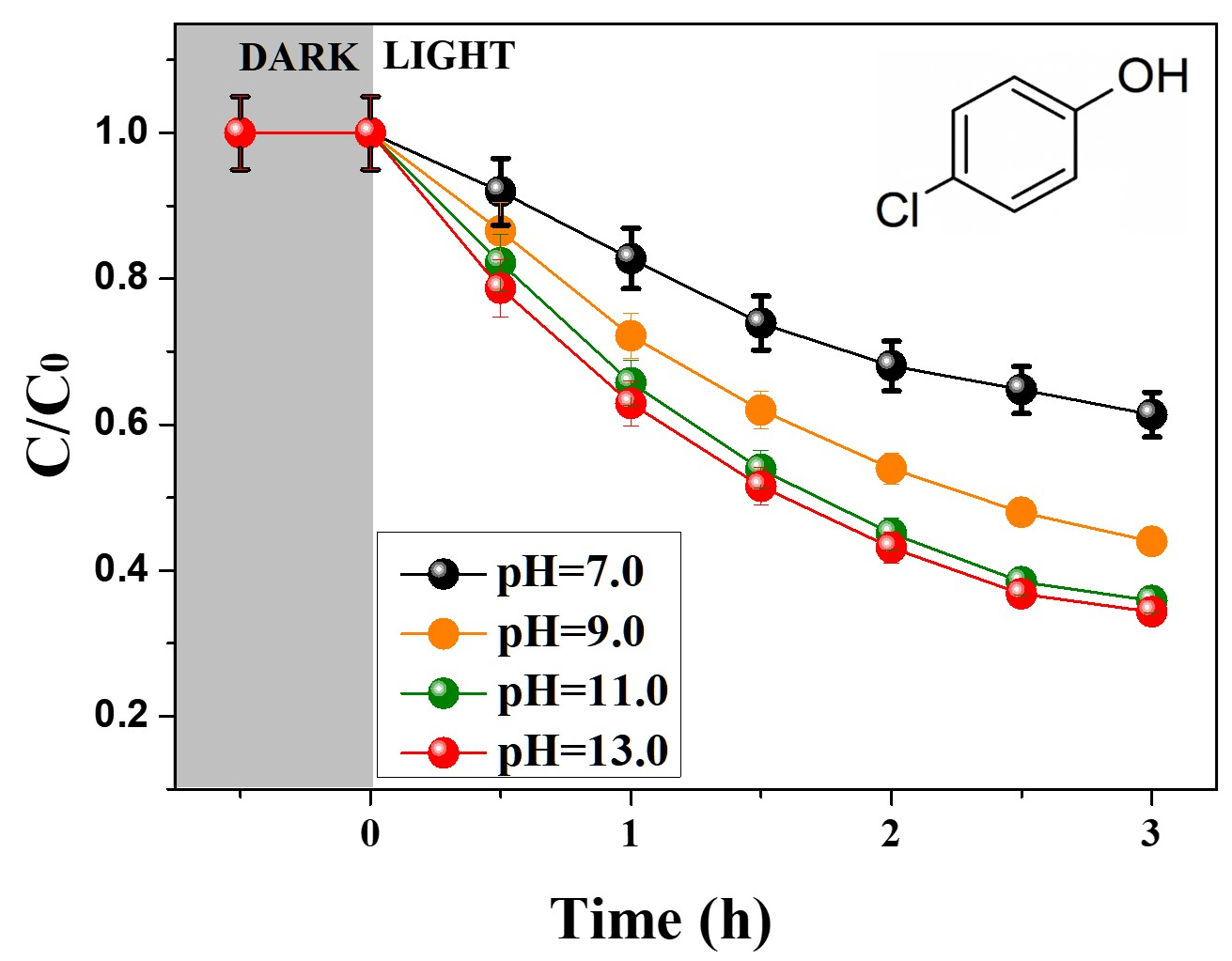
**

**Figure S7**. PCD activity of ZrO_2_–B NPs using 4-CP with or without DMPO (radical scavenger) under UV light irradiation (*λ* ≥ 225 nm).

References

S1. J. Ji, G. Zhang, H. Chen, Y. Li, G. Zhang, F. Zhang, Z. Fan, J. Mater. Chem., 21 (2011) 14498-14501.

S2. S. Yang, Y. Kim, S. Park, K.-j. Kim, H. Lee, Chem. Asian J., 6 (2011) 2362 – 2367.
